# Editorial: Game Changer - Next Generation Sequencing and Its Impact on Food Microbiology

**DOI:** 10.3389/fmicb.2018.00363

**Published:** 2018-03-09

**Authors:** Jennifer Ronholm

**Affiliations:** ^1^Department of Animal Science, Faculty of Agricultural and Environmental Sciences, McGill University, Montreal, QC, Canada; ^2^Department of Food Science and Agricultural Chemistry, Faculty of Agricultural and Environmental Sciences, McGill University, Montreal, QC, Canada

**Keywords:** foodborne pathogens, food microbiology, whole genome sequencing, next generation sequencing, metagenomics

In the decade between 2004 and 2014 the rapid evolution of next generation sequencing (NGS) platforms reduced the cost of sequencing a gigabase of nucleic acid from $1,000 to $10. This resulted in the widespread availability of DNA sequence data and started in a revolution in field of food microbiology.

A critical activity in maintaining microbiological food safety is fast and accurate outbreak detection and source attribution. Successfully delineating an outbreak relies primarily on a high-resolution comparison of the relatedness of clinical, food, and environmental samples. Traditional molecular techniques such as pulsed-field gel electrophoresis (PFGE) or multi-locus sequence typing (MLST) also rely on detecting subtle genomic differences between isolates, but neither technique is able to utilize the whole genome. Microbial whole genome sequencing (WGS) followed by detection of single nucleotide polymorphisms (SNPs) can extract relevant information from the entire genome and provides the highest-resolution examination of the relatedness of isolates possible. The genomic data that is generated in the process can also be mined for the presence of virulence factors, antibiotic resistance genes, or other genetic markers of interest. Therefore, WGS is rapidly displacing other molecular typing methods for foodborne outbreak analysis and surveillance.

Other sequencing techniques such as shot-gun metagenomics, single-cell sequencing, and 16S rRNA targeted-amplicon sequencing are routinely being used by microbial ecologists to characterize entire microbial communities. In a food microbiology context, these techniques are providing a detailed look at the microbiomes of food, food-animals, food-spoilage, fermentation, un-defined starter cultures, and probiotic products.

This e-book includes inputs from 242 authors who have used this Research Topic as a platform to discuss NGS as it relates to food microbiology including recent findings, novel applications, future directions, and concerns about the shift away from more traditional methods.

Twenty-two years ago, the first draft genome sequence of a bacteria (*Haemophilus influenzae*) was completed using a protocol that required individually Sanger sequencing the entire 1.8 million bp genome one 460 bp piece at a time. Each piece first had to be cloned into plasmid vectors and propagated in *Escherichia coli* cells (Fleischmann et al., 1995). This was a monumental task that that required generating 9,500 *E. coli* clones to obtain a single draft genome with 5x coverage. Today, most well equipped microbiology laboratories can sequence, assemble, and annotate several bacterial draft genomes in a week. A whole genome sequence can provide identification, characterization, and subtyping of a microbe for epidemiological investigations at a speed and level of precision that was previously not possible (Ronholm et al., 2016). This precision allows regulatory agencies to make connections between clinical cases that may have been overlooked using traditional techniques. For example, *Salmonella enterica*, which is one of the most prevalent foodborne pathogens in the world (Scallan et al., 2011), is divided into >2,500 serovars, only a few of which are commonly associated with human illness. There is high genetic homogeneity between isolates belonging to the same serovar. Genetic homogeneity between serovars that regularly cause illness in humans creates a problem for public health organizations. For example, the majority of clinical *Salmonella* isolates received by the New York State Department of Health are *S*. ser. Enteritidis, and of these isolates, 50% are indistinguishable by pulsed field gel electrophoresis (PFGE) (Allard et al., 2013). The result is that from serotyping or PFGE data alone there is no way of knowing if there is a single *S*. Enteritidis outbreak occurring or if several simultaneous outbreaks are co-occurring—the resolution is simply not high enough to differentiate between outbreaks. WGS solves this problem and is therefore very useful, particularly in investigating *S. enterica* outbreaks. This is perhaps why four articles in research topic are dedicated to discussing *Salmonella* in the context of WGS. The Syst-OMICS consortium (http://salmonella-systomics.ca/en/) is in the process of sequencing 4500 *Salmonella* genomes and is concurrently assessing the virulence potential of several of these isolates by cell-culture or *in vivo*. The Perspective article by Emond-Rheault et al. is the first publicly available report from this group. The intensive genomics analysis of the *Salmonella* genus presented by Emond-Rheault et al. is complemented, in this Research Topic, by the more focused article by Kovac et al. which addressed the genomics of a single serovar, *S*. Cerro. However, both articles reminded readers of the importance of correlating clinical outcome or wet-lab pathogenicity data with genomic data. Genetic mutations can lead to novel pheno- and patho-types, and studies that work through the process of gathering these complementary datasets provide invaluable information so that eventually accurate microbial risk assessments can be performed based only on genomic data.

Culture-independent diagnostic tests (CIDTs), which use nucleic acid and antigen-based assays, are beneficial to clinicians since they decrease diagnostic testing times and may even help in diagnosing cases that would have been overlooked (Huang et al., 2016). However, these techniques may be detrimental to public health, since without either an isolate or a whole genome sequence, the ability public health agencies to link clinical cases of a disease to each other and a source is severely limited. The review by Forbes et al. discussed metagenomics as a culture-independent solution to the public health short-falls of CIDTs in general, but reminded readers there are still challenges including generation non-target data, the inability to distinguish between live and dead bacterial cells, and the difficulty of assigning detected virulence and antibiotic resistance genes to a particular bacterial species. Single-cell metagenomics, still in the early stages of development, may solve the latter problem. Indeed, in this Research Topic, Yao et al. used a single-cell amplification technique to successfully analyze the metagenome of koumiss, a traditional fermented dairy product.

The article by Nieuwenhuijse et al. suggested that since metagenomics can simultaneously can detect all viruses from complex microbial sample this technique could be useful in viral surveillance. However, Nieuwenhuijse et al. also acknowledged that there are challenges with metagenomic viral detection including the excessive generation of non-target data and that the poor understanding of the virome makes data interpretation difficult. Despite these challenges, Nasheri et al. developed a technique that uses non-specific amplification of viral RNA, directly from patient fecal samples, to generate whole genome Norovirus sequences—a technique that could eventually be valuable for reconstructing transmission directionality.

NGS techniques have also allowed microbial ecologists to explore, compare, and characterize mixed microbial communities. Since ~98% of the bacteria in an environmental sample cannot be grown in the laboratory using routine techniques, this is really the first time these populations have been able to be effectively studied (Wade, 2002). The range of effects that a microbial community can have food-production, -safety, and -quality are essentially limitless. In this Research Topic four papers addressed a wide-range of microbiome related topics. NGS techniques have led to numerous recent studies into the effect of bacteria on the final sensory properties of wines. The review by Belda et al. introduced readers to influence of the soil microbiome on wine-quality, while Morgan et al. summarized the findings of several studies investigating the effects of the grape and vine microbiomes. The paper by Best et al. investigated the gastrointestinal microbiome of the Pekin Duck and while the data presented in this article provided only baseline information on the normal microbiome for this food animal, the author's also note that that there is potential for the microbiome of food animals to be optimized in the future to improve growth yield and exclude pathogens. The study by Morovic et al. used 16S rRNA sequencing to characterize a variety of probiotic supplements and discovered differences in both the classification and abundance of bacteria between the supplement and the bacteria named on the label. This article draws attention to the possible uses of NGS in detecting adulteration in probiotic supplements—which, given their findings, may be an important topic to address. While culture-independent sequencing techniques are critical for understanding the mixed microbial communities present in a food item, food-producing animal, or a probiotic supplement, the mini-review by Gill made the important point that the majority of microorganisms that are relevant to food-microbiology are easily cultured, and that genomics complements rather than replaces culture-based analysis when addressing most microbiological issues surrounding food production and distribution.

Despite the availability of NGS techniques and the amazing potential that sequencing data has in food safety management a few articles also reminded observant readers that there are still hurdles to be overcame before the full implementation of this technology by regulatory bodies. The perspective article by Griffiths et al. introduced readers to the importance of metadata in understanding the context of whole genome sequences. Griffiths et al. informed readers that there are several important issues with metadata regarding language and consistency in major genomic databases and introduced us to ontologies—hierarchies of well-defined and standardized vocabularies interconnected by logical relationships, as a possible solution to this problem. To get the most from WGS for outbreak detection and surveillance global cooperation and data sharing is required. However, as noted in Taboada et al. several jurisdictions are hesitant about the rapid release of genomic data to public archives; and clarifying issues surrounding sensitives of metadata, legal implications of increased source attribution accuracy, and intellectual property rights surrounding sequence data will be important in moving forward.

There were a few important topics related to both NGS and food microbiology that were not addressed in this issue. The most glaring omission is likely the exclusion of any mention of foodborne parasites. The further development of metagenomics has a huge potential for the detection, surveillance, and characterization of parasites, most of which are not culturable in the laboratory. In addition, a closed genome sequence has yet to be completed for most foodborne parasites, which is a goal several groups are actively perusing. The Research Topic was limited to DNA sequencing and failed to address the developing field of RNA-seq including its use as research tool, and its potential to help solve some of the existing issues with metagenomics for CIDT. Lastly, although researchers in the field of food microbiology openly discuss the difficulty they experience in recruiting highly qualified personnel in bioinformatics and microbial genomics and a few articles in this Research Topic articles alluded to this need, a study to systematically assess and quantify this need has yet to be performed and would be valuable to see in the future.

Overall the collection of articles in this Research Topic presents several of the benefits that embracing NGS would bring to the field of microbiology, while warning that, at least for now, genomic data complements rather than replaces *in vivo* virulence assays and culture-based studies of physiology. Several of issues surrounding data sharing, archiving, and analysis were also raised. I hope that research topic adequately informs readers about the benefits that NGS offers to the field of food microbiology and about the many challenges that have yet to be overcome in this field.

## Author contributions

The author confirms being the sole contributor of this work and approved it for publication.

### Conflict of interest statement

The author declares that the research was conducted in the absence of any commercial or financial relationships that could be construed as a potential conflict of interest.
